# Types of Punishment and Self-Reported Willingness to Change Behavior: Chain Mediation by Perceived Motivation for Punishment and Trust, and the Moderating Role of Group Relationship

**DOI:** 10.3390/bs16081450

**Published:** 2026-08-21

**Authors:** Zhen Zhang, Li Kong, Chunhui Qi

**Affiliations:** Faculty of Education, Henan Normal University, Xinxiang 453007, China; zhangzhen201912@htu.edu.cn (Z.Z.); 2018224004@stu.htu.edu.cn (L.K.)

**Keywords:** willingness to change behavior, type of punishment, perception of punishment motivation, trust

## Abstract

Correcting student misconduct is a central issue in educational psychology and classroom management; teachers often use punishment to correct students’ inappropriate behavior. Numerous empirical studies have shown that punishment influences individuals’ willingness to change their behavior, but the underlying mechanisms remain to be elucidated. This study employed a scenario simulation method to survey 240 middle school students, aiming to explore the relationship between types of punishment and students‘ self-reported willingness to change their behavior in response to hypothetical scenarios. Furthermore, the study examined the chained mediating effects of perceived punishment motivation and trust, as well as the moderating effect of group relationship. The results showed that perceived punishment motivation and trust exert a chained mediating effect between punishment type and self-reported willingness to change behavior. Punishment first is associated with enhanced perception of the prosocial motivation behind the punishment, which in turn is associated with higher trust in the punishing teacher, and ultimately with greater self-reported willingness of misbehaving students to change their behavior; that is, perception of punishment motivation and trust sequentially transmit the effects of punishment. Furthermore, this transmission process is moderated by group relationship: when the punisher is a teacher (in-group), the influence of punishment type on students’ self-reported willingness through perceived punishment motivation and trust is stronger, whereas this effect is not significant when the punisher is an AI (out-group). These findings reveal the psychological pathway through which punishment influences self-reported willingness to change behavior in a hypothetical scenario via sequential processing of cognitive attribution (perceived punishment motivation) and attitudinal evaluation (trust), and highlight the moderating role of group relationship in this process.

## 1. Introduction

Correcting student misconduct is a central issue in educational psychology and classroom management. The Theory of Planned Behavior posits that an individual’s behavioral intention reflects their propensity to make behavioral decisions and plays an active role in driving the individual to ultimately make a specific behavioral decision. Behavioral intention is influenced by the individual’s attitude toward the behavior, subjective norms, and perceived behavioral control, with attitude stemming from the individual’s cognitions and beliefs regarding the outcomes of the behavior (Ajzen, 1991; Fishbein & Ajzen, 1977). Intention refers to the willingness to perform a certain behavior; it describes the likelihood that a person believes they will engage in a specific behavior. This study draws on the theoretical logic of the Theory of Planned Behavior, “cognition to attitude to behavioral intention”, namely, that an individual’s cognitive evaluation of a particular object influences their attitude, which in turn influences their willingness to change behavior, to construct the chain mediation model for this study. Research has found that whether an individual expresses a willingness or intention to engage in a particular behavior can directly predict behavioral outcomes (Kollmuss & Agyeman, 2002). Conner and Norman argue that a key factor influencing behavioral change is behavioral intention, defined as a self-instruction or decision to act, reflecting the underlying motivation or commitment to carry out the action. Intentions are categorized into goal-oriented intentions (e.g., “I intend to become better”) and behavior-oriented intentions (e.g., “I will make changes every week to become better”) (Conner & Norman, 2025). Therefore, this study classifies the willingness to change behavior into the willingness to change current non-compliant behavior and the willingness to comply with behavioral norms in the future. Michaelsen and Esch comprehensively integrated a behavioral change model, which proposes that the behavioral change process involves three motivational states (approach motivation, avoidance motivation, and certainty motivation), where avoidance motivation refers to physiological and behavioral changes generated by individuals to escape punishment or potential harm (Michaelsen & Esch, 2023). Broadly speaking, behaviorism assumes that all aspects of an individual’s behavior develop through experiences related to environmental stimuli and responses to those stimuli (Heimlich & Ardoin, 2008). Accordingly, this study will explore, based on the Theory of Planned Behavior, the mechanisms through which punishments for rule-breaking influence students’ willingness to change their behavior.

### 1.1. Perception of Punishment Motivation as a Mediator Between Punishment Type and Trust

Skinner’s theory of reinforcement conceptualizes punishment as an external consequence that reduces the occurrence of undesirable behavior (Skinner, 1965). Over time, the concept of punishment has transcended its behaviorist roots and taken on more complex, and at times even subtle, forms. In this study, punishment refers to disciplinary actions imposed on students who violate rules by authority figures (teachers) or institutional systems (AI behavior management systems) with institutional power, rather than informal sanctions among peers based on social norms (Douglas et al., 2024). In the experimental context of this study, punishment was operationalized as a formal disciplinary decision made by a teacher or an AI system in accordance with school rules and regulations (i.e., making a public self-criticism in class), with a clear source of institutional authority and formal enforcement procedures. Furthermore, this study posits that punishment, as a type of stimulus, can form a “misbehavior–punishment” association by presenting aversive consequences linked to rule-breaking, thereby Increasing the motivation of students who have violated rules to change their behavior (Heffernan, 1988; Skiba & Peterson, 1999). Simultaneously, it heightens students’ awareness of rules and stimulates an initial willingness to change their behavior (Radkani et al., 2025). Empirical research indicates that moderate, standardized punishment can clearly convey the message that “rule-breaking is unacceptable,” helping students establish clear behavioral boundaries and further motivating them to comply with rules (Cui et al., 2017). From the perspective of social norms, social sanctions are an important means of upholding normative social norms. When individuals punish others through social sanctions, this can promote prosocial behavior, which in turn fosters a willingness to change one’s own behavior (Carlsmith et al., 2002). In the field of management, research indicates that employees’ attitudes and behaviors are significantly influenced by feedback from colleagues or supervisors, whether through praise or criticism (Ali et al., 2014; Kuhnen & Tymula, 2012). In psychological studies, researchers have used signaling game tasks to find that both social punishment and monetary punishment can suppress individuals’ deceptive behavior (Yuan et al., 2025).

Research has shown that the manner in which punishment is administered influences an individual’s internal cognitive processes, which in turn affect the individual’s attitudes and behaviors (Podsakoff et al., 2006). Fishbein proposed the “cognition–affect–behavior model,” which emphasizes that an individual’s intention and willingness to exhibit a certain behavior are governed by their attitude toward that behavior (affection), and that this attitude is shaped by their cognitive perceptions and views regarding the execution of that behavior (cognition) (Fishbein & Ajzen, 1977). In this context, cognition refers to an individual’s evaluation and understanding of a particular behavior (such as motivation or fairness), while emotion represents an attitudinal response to perceptions and cognition (such as support or trust). Attribution theory suggests that individuals tend to seek causes for the outcomes of their own or others’ behaviors, and these attributions influence subsequent attitudes and behaviors (Harvey & Weary, 1984). Therefore, the effectiveness of punishment does not depend on the punishment itself, but rather on the individual’s interpretation of the motives behind it. Some researchers have proposed the concept of prosocial punishment motivation from the perspective of social relationships, arguing that the purpose of punishment is to restore positive relationships among the offender, the victim, and the groups to which they belong. Furthermore, if punishment is administered appropriately, it can convey a message of social inclusion (van Prooijen et al., 2008), thereby facilitating the offender’s reintegration into the group. Being punished triggers a cognitive process in which the offender spontaneously asks, “Why is the other person punishing me?” If the punisher demonstrates fairness and clear objectives, students are more likely to perceive prosocial motives behind the punishment. When individuals perceive prosocial motives, they view the punisher as well-intentioned and reliable, thereby fostering trust; if antisocial motives are perceived, suspicion and resistance arise, preventing the formation of trust (Gollwitzer & Okimoto, 2021), which in turn reduces identification with the rules and the willingness to change voluntarily (de Vel-Palumbo et al., 2023). Therefore, this study proposes:

**Hypothesis** **1.** *The type of punishment indirectly influences students’ trust in teachers through the perception of punishment motives.*

### 1.2. The Chain Mediation of Perceived Motivation for Punishment and Trust

Trust is central to emotional bonds in interpersonal interactions; it refers to the willingness of individuals to entrust social resources to others when they lack sufficient information to judge their own motives and behaviors. As a lubricant for social systems, trust can facilitate cooperative behavior and is a key prerequisite for the effectiveness of behavioral interventions (Wang & Murnighan, 2017; Kondo et al., 2021). During interactions with others, individuals can assess whether others are trustworthy through various cues, such as personal traits and behavioral patterns (e.g., helping and punishing) (Siddique et al., 2022). Research has found that observers trust third parties who punish rule-breakers more than those who do not (Kupfer & Tybur, 2023). When teachers impose punishments on students who violate classroom rules, such as suspending them from class or sending them to the office, students perceive this as a sign of “responsibility and concern for their growth,” leading them to view the teacher as a legitimate and trustworthy authority figure (Gregory & Ripski, 2008). Social cognitive theory posits that individuals are more willing to accept influence from those they trust, are willing to follow the rules and expectations conveyed by them, and consequently are willing to change one’s behavior to meet the expectations of those who trust them (Bandura, 1986). When individuals trust the punisher, they perceive the punishment as reasonable and beneficial, leading them to voluntarily adjust their behavior; conversely, if trust is absent, individuals will reject the punishment even if it is reasonable (Yamagishi et al., 2015). Research by Sun et al. confirms that third-party punishment can enhance individuals’ trust in the punisher, and that the level of trust directly determines an individual’s willingness to cooperate and change behavior (Sun et al., 2023). Therefore, this study proposes:

**Hypothesis** **2.** *The type of punishment indirectly influences students’ willingness to change behavior: (a) Willingness to change current non-compliant behavior, and (b) willingness to comply with behavioral norms in the future through perceived punishment motivation and trust.*

### 1.3. The Moderating Role of Group Relationship

Individuals form conceptually rich social categories as early as childhood, defining themselves and others in terms of groups, and their group membership becomes a key factor influencing their attitudes and behaviors (Liberman & Shaw, 2018). This classification process also occurs in educational contexts. With the continuous development of educational technology, AI-powered behavioral management systems are increasingly assuming the roles of supervisors and adjudicators. In this study, punishers were categorized into two groups: human teachers and AI-powered behavioral management systems that record student disciplinary data and make disciplinary decisions based on school regulations. When an AI system acts as a “disciplinary decision-maker,” do students exhibit psychological reactions based on group affiliation toward it, just as they do toward teachers? Computers as social actors theory (CSAT, Nass et al., 1995) states that when technological systems exhibit sufficient social cues (such as language and role identities), humans unconsciously and automatically treat them as they would human social partners. Self-categorization theory divides self-categories into three levels: the highest level focuses on the distinctions between humans and other species; the middle level focuses on the distinctions between one’s own in-group and out-groups; and the lowest level focuses on the distinctions between an individual, as a member of a group, and other members within that group (Turner & Reynolds, 2012). Under the CSAT paradigm, researchers argue that established social psychological mechanisms, such as in-group preference, stereotypes, and social categorization, are equally applicable to human–AI interactions (Nass & Moon, 2000; Shen & Wang, 2021). Specifically, individuals engage in social categorization during human–AI interactions, classifying robots or AI systems as members of the in-group or out-group. The theory of implicit bias (Amodio & Devine, 2005; Turel & Kalhan, 2023) posits that people generally harbor implicit biases against artificial intelligence, which manifest as aversion to AI. As prejudice often develops toward out-groups to protect dominant in groups (Hamby et al., 2024), we argue that people tend to automatically classify AI as an out-group, similar to how they classify other marginalized groups, such as racial or sexual minorities. Furthermore, some research suggests that machines are not human and never will be. This is because, as things stand, the agent possesses its own personal identity, grounded in the social identities that other agents—including people and machines—recognize, rather than the designer’s craft or the user’s will. Achieving this would require that the agent has a level of social intelligence and a capacity to interact with the world that does not yet exist. Therefore, no AI agent belongs to the superordinate category of “human” (Seaborn, 2025). Moreover, research from the perspective of “speciesism” points out that people believe humans possess higher moral value; since AI lacks human biological attributes (such as a physical body and vital signs) and a “true self,” it is naturally viewed as an “outsider” and an “out-group.” Even if AI is human-like in appearance or capability, people will still prioritize humans because “AI is not Homo sapiens,” thereby denying AI’s “in-group identity” (De Freitas et al., 2023). Existing research has found that treating robots as group members leads to a decline in in-group identity, and the more robots there are in a group, the lower the in-group identity becomes (Savela et al., 2021). Therefore, this study argues that although an AI-powered behavioral system acts as a decision-maker in the imposition of penalties, it still constitutes an out-group distinct from humans. But it is important to clarify that this study’s operationalization of group relationship is grounded in the most fundamental dimension of social categorization—the human–non-human distinction—as outlined by self-categorization theory (Turner & Reynolds, 2012). We do not claim that this single dimension captures all differences between teachers and AI systems. Rather, we position our work within the broad framework of social categorization and interpret our findings as consistent with the predictions derived from this framework. Social identity theory posits that in intergroup relationship, individuals exhibit in-group favoritism, meaning they evaluate in-group members positively while evaluating out-group members negatively (Cikara & Van Bavel, 2014). Research has found that when punishment occurs within an in-group context, people tend to interpret it as a benevolent act aimed at upholding group norms, due to the shared identity and emotional bonds among in-group members (McAuliffe & Dunham, 2016). Since shared group identity provides the cognitive foundation for the “benevolence assumption,” this perception of benevolent, prosocial motives for punishment is more likely to translate into trust in the punisher. In studies on punishment and compliance, individuals exhibit higher baseline trust in in-group authorities, and punishment has a stronger effect on promoting behavioral change (Hao & Xu, 2023). Signal theory posits that punishment is essentially a social signal, it conveys information about the punisher’s motives, capabilities, and values (Jordan et al., 2016). However, different group relationship alter how rule-breakers interpret punitive behavior: actions by in-group members are more likely to be perceived as benevolent, whereas those by out-group members are viewed as threatening (Z. Zhang et al., 2020). In summary, this study proposes Hypotheses 3 and 4.

**Hypothesis** **3.** *Group relationship moderates the indirect effect of punishment type on trust, such that when the punisher is an in-group member (teacher), the positive effect of punishment type on trust via perceived punishment motivation is stronger.*

**Hypothesis** **4.** *Group relationship moderates the chained indirect effect of punishment type on willingness to change behavior (willingness to change current rule-breaking behavior and willingness to comply with behavioral norms in the future), such that when the punisher is an in-group member (teacher), the positive effect of punishment type on students’ willingness to change behavior through perceived punishment motivation and trust is stronger.*

**Figure 1:**
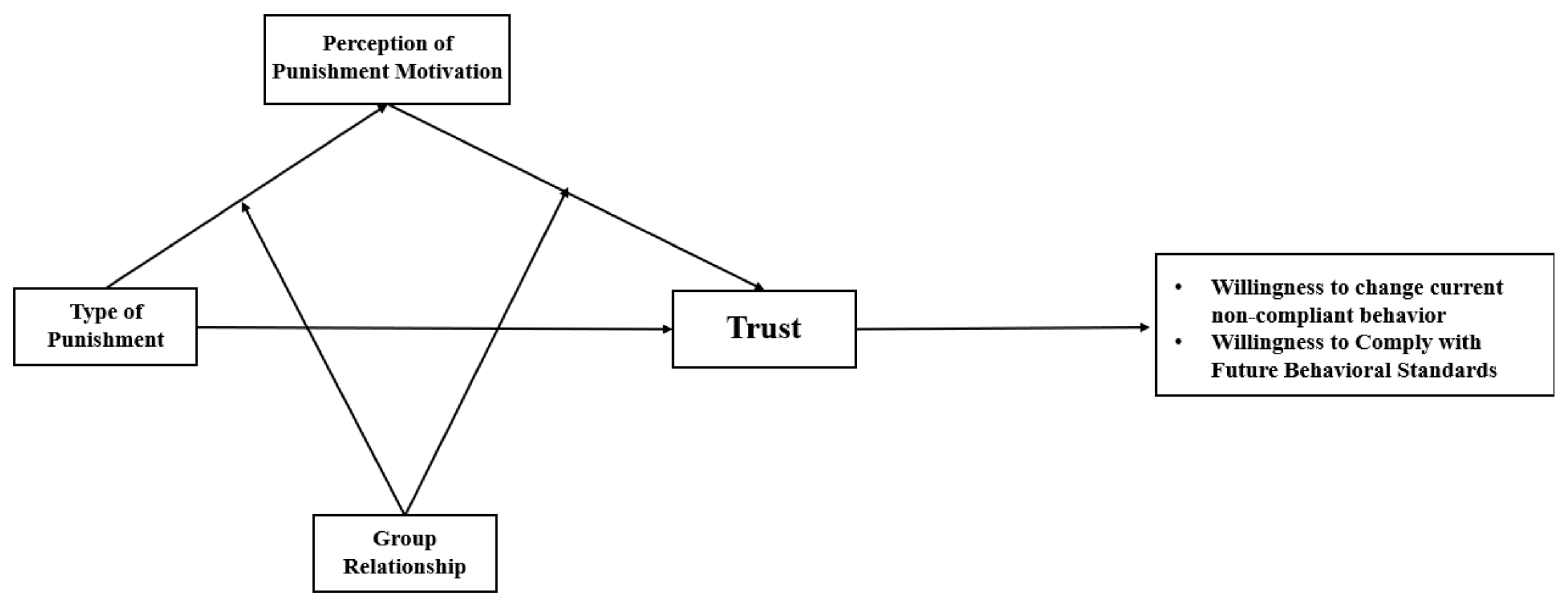
Research Model Diagram.

Although punishment, as an external stimulus, can directly trigger students’ willingness to change their behavior, its effects are not achieved through a single mechanical reflex; cognitive and attitudinal responses elicited by the stimulus may also play a mediating role (Podsakoff et al., 2006). Specifically, this study posits that students’ cognitive attributions regarding the motives behind punishment (i.e., perceived punishment motives) constitute a key cognitive mediating pathway, determining whether students interpret punishment as benevolent guidance aimed at promoting growth or merely as a means of control; simultaneously, the trust attitude toward the punisher formed based on perceived punishment motives serves as an affective mediator, further influencing students’ acceptance of punishment. The combined effect of these two mechanisms transforms the external stimulus of punishment into individuals’ internal willingness to change current rule-breaking behavior, which in turn influences their willingness to comply with behavioral norms in the future. In summary, the conceptual model proposed in this study is illustrated in Figure 1.

## 2. Research Methods

### 2.1. Participants

Prior to data collection, this study referred to the classic sample size recommendations for mediation effects by Fritz and MacKinnon (2007), a study that, through large-scale Monte Carlo simulations, provided the minimum sample sizes required to test for mediation effects under various combinations of effect sizes. According to their findings, under conditions of moderate effect sizes (α = β = 0.39), a simple mediation model requires at least approximately 162 participants to achieve 0.8 statistical power (Fritz & MacKinnon, 2007). In addition, we used G*Power 3.1 software to calculate the sample size required for the simplified path model, and the results showed that at least 146 participants are required to achieve 85% statistical power. To account for potential invalid responses (e.g., failure to attend to the task), we oversampled to include approximately 260 participants. The participants were selected from seventh-grade students at a junior high school in a city in Henan Province. A total of 260 questionnaires were distributed, and 240 valid responses were collected, resulting in a response rate of 92.30%. Among them, 119 were male, accounting for 49.60%, and 121 were female, accounting for 50.04%. The average age was 13.35 years.

### 2.2. Experimental Design

A 2 (punishment type) × 2 (group relationship) between-subjects experimental design was employed. Punishment types were categorized as “punishment” and “no punishment”; group relationship was categorized as “in-group” (teachers) and “out-group” (AI). The dependent variable was willingness to change behavior, including willingness to change current rule-breaking behavior and willingness to comply with behavioral norms in the future, with perceived punishment motivation and trust serving as mediating variables. The independent variable (punishment type) and the moderator (group relationship) were operationalized using simulated scenario stories. Four distinct experimental conditions were designed to manipulate the presence or absence of punishment and different group relationships.

### 2.3. Experimental Materials and Tools

#### 2.3.1. Punishment Scenario

Adapted from the student campus criticism scenario designed by Cheng et al. (2025), using a scenario involving a teacher’s disciplinary action as an example: Hello, classmate. Please imagine the following scenario: Today it was your turn to be on duty, responsible for cleaning the classroom. However, because you were in a hurry, you forgot to dust the windowsills, and some paper scraps were left on the floor. As a result, the Student Council officer who inspected the classroom deducted points from your class. Disciplinary matters in your class have always been handled by your experienced homeroom teacher, Mr./Ms. Li. With 10 years of experience leading a class, Mr./Ms. Li has dealt with various student disciplinary issues. Your record of failing to meet cleaning standards has been reported to Mr./Ms. Li, who will make the final decision on how to handle the matter based on class rules and your past conduct. See Appendix A for all materials.

#### 2.3.2. Manipulation Check

One question was designed to test the effectiveness of manipulating the type of punishment, and another to test the effectiveness of manipulating group dynamics. The test questions were as follows: Given a scenario, select from the options whether you were punished and whether the punishment was administered by a teacher or an AI system.

#### 2.3.3. Perceived Punishment Motivation Scale

The measure of perceived punishment motivation was adapted from the scale developed by de Vel-Palumbo et al. (2023), which divides punishment motivation into five dist it can be retained inct dimensions. This study selected the subscale measuring prosocial punishment motivation. It consists of four items designed to assess prosocial punishment motivation, such as “Helping me become a better member of the group” (1 = Strongly Disagree, 5 = Strongly Agree; Cronbach’s α = 0.76). Higher scores indicate a higher level of perceived prosocial punishment motivation.

#### 2.3.4. Trust Scale

Adapted from the scale of human–AI trust developed by Yokoi and Nakayachi (2021). The scale consists of three items, such as “Teacher Li/the AI system is trustworthy” (1 = Strongly disagree, 5 = Strongly agree; Cronbach’s α = 0.78). A higher score indicates a higher level of trust in the teacher/AI system among students.

#### 2.3.5. Behavior Change Intention Scale

This scale was adapted from scales on behavior change (current rule-breaking behavior and future behavior) developed by de Vel-Palumbo et al. (2023) and Mott and Solomon (2025), among others. This study employs a scale consisting of a total of 7 items across two dimensions: 5 items measure willingness to change current rule-breaking behavior, such as “I do not intend to repeat my mistakes” (Cronbach’s α = 0.91); and 2 items measure willingness to comply with behavioral norms in the future, such as “Ten years from now, I will not engage in the same behavior” (Cronbach’s α = 0.78). The total scale uses a five-point rating scale (1 = Strongly Disagree, 5 = Strongly Agree; Cronbach’s α for the total scale = 0.93). Higher scores indicate a stronger willingness among students to change their rule-breaking behavior.

### 2.4. Experimental Procedure

Before the experiment began, the homeroom teacher’s representative distributed the “Parental Informed Consent Form” to the parents. After the parents gave their consent, we distributed the “Student Informed Consent Form” to the students. Once the students gave their consent, we began administering the questionnaire. All participants completed the experiment using a paper-and-pencil questionnaire. Participants first provided their basic personal information (gender, age, grade level), then read an identical scenario description regarding a violation of duty-roster rules, and subsequently, depending on their condition assignment, read different penalty feedback messages; after reading the feedback, participants were asked to answer three questions in sequence: (1) Whether they were punished in the scenario (yes or no); (2) Whether the punisher in the scenario was a teacher or an AI (yes or no); (3) Whether they believed they deserved to be punished (yes or no). After completing the operational test questions, participants first rated their perception of the teacher’s or AI system’s motivation to punish in the scenario, followed by a trust rating, and finally a rating of their own willingness to change their behavior. All items used a 5-point Likert scale.

## 3. Results

### 3.1. Manipulation Check

In this study, a task to verify condition recognition was included to ensure that participants had correctly read the condition information. Chi-square tests revealed that the vast majority of participants were able to correctly identify the condition they were in. Under the punishment condition, 98.3% of participants (114/116) selected “yes”; under the no-punishment condition, 98.4% of participants (121/123) selected “No.” The chi-square test revealed a significant association between condition and response, χ^2^(1, 240) = 220.42, *p* < 0.001, indicating that the manipulation was successful. Participants were asked to answer “Was it the teacher or the AI who punished you?” (Teacher/AI). Under the teacher condition, 98.3% of participants (118/120) correctly selected “teacher”; under the AI condition, 97.5% of participants (117/120) correctly selected “AI.” The chi-square test results were significant, χ^2^(1, 240) = 175.22, *p* < 0.001, indicating that the manipulation was effective.

### 3.2. Correlation Analysis Among Variables

A correlation analysis was conducted on punishment type, group relationship, perceived punishment motives, trust, and willingness to change behavior; the results are shown in Table 1. Punishment type was significantly negatively correlated with perceived punishment motives, trust, and willingness to change behavior (*r* = −0.17, *p* < 0.01; *r* = −0.52, *p* < 0.01; *r* = −0.29, *p* < 0.01). Group relationship was not significantly correlated with punishment type, perceived punishment motives, or trust (*r* = −0.05, *p* > 0.05; *r* = 0.03, *p* > 0.05; r = −0.11, *p* > 0.05), but was significantly negatively correlated with willingness to change behavior (*r* = −0.39, *p* < 0.01). Perceived punishment motivation was significantly positively correlated with trust (*r* = 0.45, *p* < 0.01). Trust was significantly positively correlated with willingness to change behavior (*r* = 0.28, *p* < 0.01).

### 3.3. Analysis of Variance

**Figure 2:**
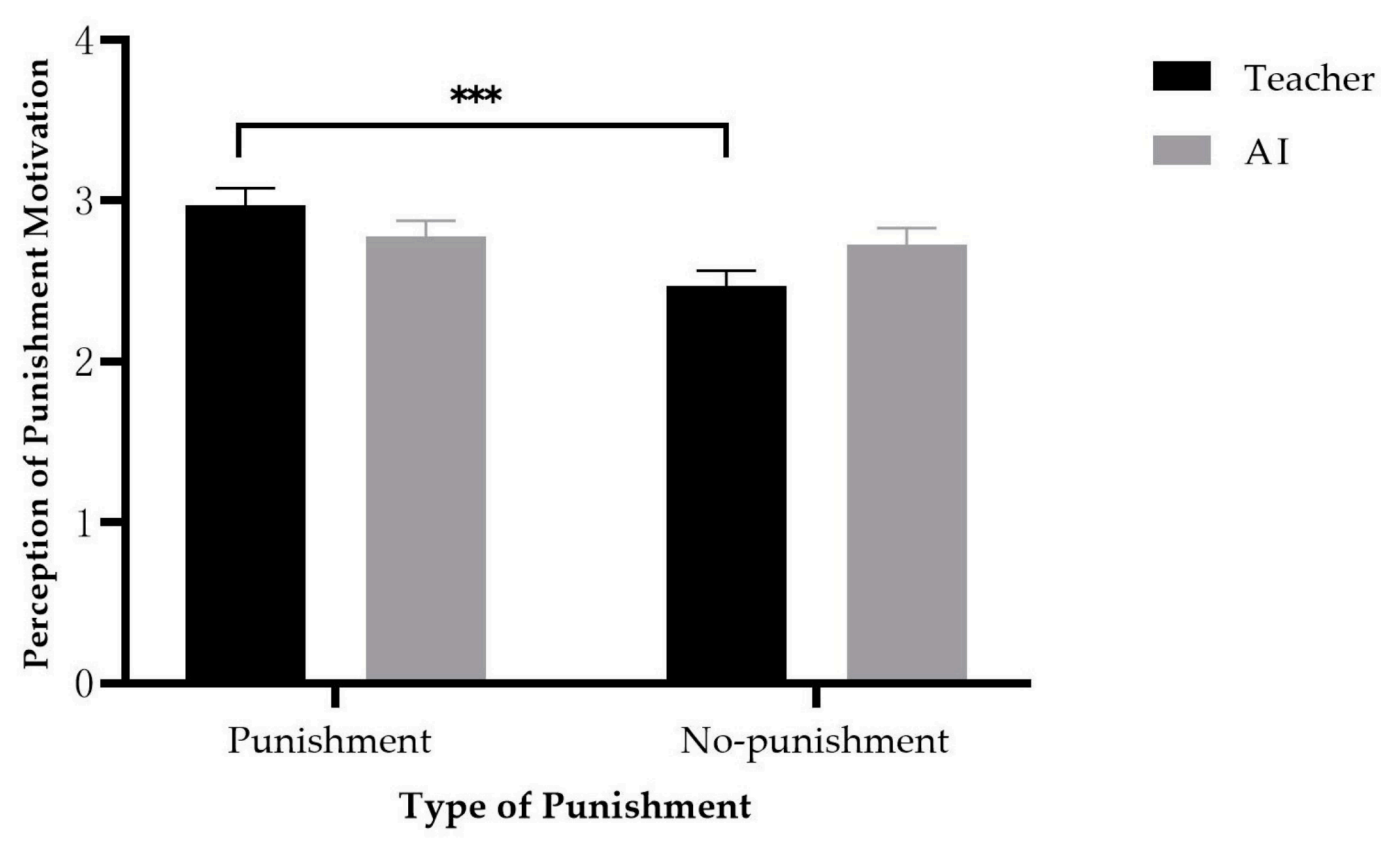
Mean Perceived Motivation for Punishment Across Different Group Relationships. *** *p* < 0.001.

The results of a 2 (punishment type: punishment, no-punishment) × 2 (group relationship: in-group, out-group) between-subjects analysis of variance were obtained, with punishment type and group relationship as independent variables and perceived punishment motivation as the dependent variable. The results show, The main effect of punishment type reached statistical significance. (*F*(1, 236) = 7.46, *p* < 0.01); students perceived significantly higher prosocial punishment motives in the punishment condition than in the no-punishment condition, indicating that punishment is more likely to prompt students to perceive teachers’ prosocial punishment motives than the absence of punishment; The main effect of group relationship was not significant, *F*(1, 236) = 0.09, *p* > 0.05. The interaction between punishment type and group affiliation reached statistical significance, *F*(1, 236) = 5.18, *p* < 0.05. Further simple effects analysis revealed that when the punisher was a teacher, the level of prosocial punishment motivation perceived by students in the punishment condition (*M* = 2.97, *SD* = 0.11) was significantly higher than that perceived in the no-punishment condition (*M* = 2.46, *SD* = 0.09). However, when the punisher was an AI, the difference between the two conditions was not significant, as shown in Figure 2.

### 3.4. Analysis of Mediating Effects

Based on the results of the correlation analysis, we examined the separate mediating effects of perception of punishment motivation and the chained mediating effects of perception of punishment motivation and trust, using frequency of punishment and familiarity with class rules as control variables. We conducted hierarchical regression analysis on each variable using dummy coding. The regression results were used to determine whether the conditions for testing the mediation effect were met. To verify the sole mediating effect of perception of punishment motivation, SPSS 27.0 was used to obtain confidence intervals for the indirect effect via the bootstrap method, and PROCESS Model 4 was employed for the testing. To verify the chained mediating effect of perception of punishment motivation and trust, PROCESS Model 6 was employed for testing.

#### 3.4.1. The Independent Mediating Effect of Perceived Punishment Motives

**Figure 3:**
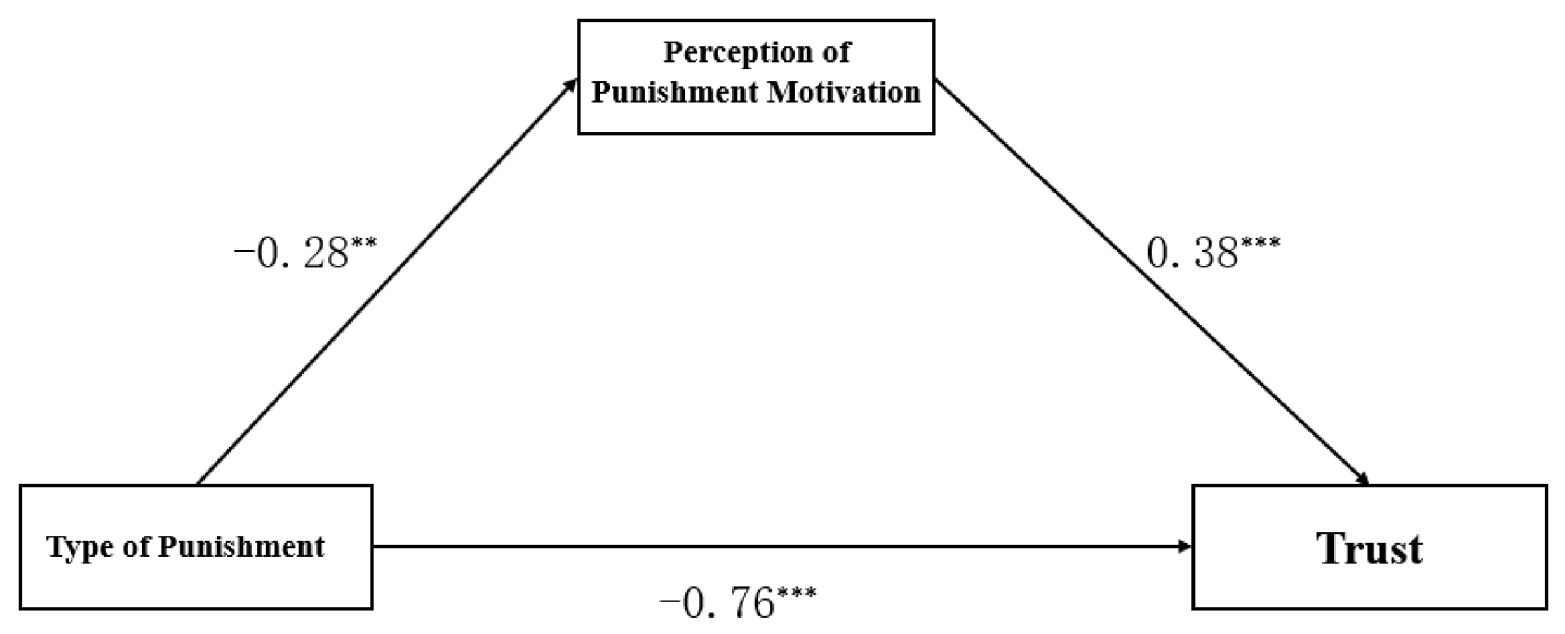
Path Diagram of the Mediator Model. ** *p* < 0.01, *** *p* < 0.001.

The results of the mediation analysis are presented in Table 2. The results indicate that the type of punishment significantly and negatively predicts trust (*β* = −0.87, *p* < 0.001) and significantly and negatively predicts perceived punishment motivation (*β* = −0.28, *p* < 0.01); perceived punishment motivation significantly and positively predicts trust (*β* = 0.38, *p* < 0.001). The total effect of punishment type on trust is significant, *effect* = −0.87 (95% CI = [−1.05, −0.69]), which does not include 0; the direct effect is significant, *effect* = −0.76 (95% CI = [−0.93, −0.59]), which does not include 0; the indirect effect was significant, *effect* = −0.11 (95% CI = [−0.20, −0.02], excluding 0). The partial mediating effect of perceived punishment motivation between punishment type and trust reached statistical significance, consistent with the hypothetical model proposed in this study, as shown in Figure 3. This provides support for Hypotheses 1.

#### 3.4.2. The Chain Mediation Effect of Perceived Punishment Motivation and Trust Between Punishment Type and the Willingness to Change Current Violative Behavior

**Figure 4:**
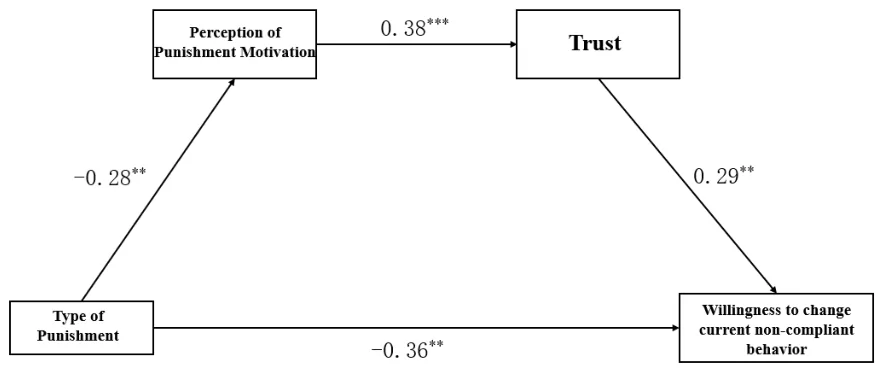
Path Diagram of the Chain-Mediated Model. ** *p* < 0.01, *** *p* < 0.001.

The results of the chain mediation analysis are presented in Table 3. The results indicate that the type of punishment significantly and negatively predicts the willingness to change current rule-breaking behavior (*β* = −0.36, *p* < 0.01) and significantly and negatively predicts the perceived motivation for punishment (*β* = −0.28, *p* < 0.01); perceived punishment motivation significantly and positively predicts trust (*β* = 0.38, *p* < 0.001); and trust significantly and positively predicts the willingness to change current rule-breaking behavior (*β* = 0.29, *p* < 0.01). The total effect of punishment type on willingness to change current rule-breaking behavior was significant, *effect* = −0.57 (95% CI = [−0.80, −0.33], excluding 0); the direct effect was significant, *effect* = −0.37 (95% CI = [−0.80, −0.34], excluding 0); the indirect effect of the path Punishment Type → Perception of Punishment Motivation → Trust → Willingness to Change Current Non-Compliance was significant, *effect* = −0.03 (95% CI = [−0.07, −0.01], excluding 0). The magnitude of this indirect effect was relatively modest. This suggests that the sequential pathway from punishment type to willingness to change behavior through perceived punishment motivation and trust, while reliable, accounts for only a small portion of the total variance in the dependent variable. This indicates that the chain mediation effect of perception of punishment motivation and trust between punishment type and current willingness to change rule-breaking behavior has reached a statistically significant level, as shown in Figure 4. This provides support for Hypothesis 2a.

#### 3.4.3. The Chain Mediation Effect of Perception of Punishment Motivation and Trust on the Relationship Between Punishment Type and Future Willingness to Comply with Behavioral Norms

**Figure 5:**
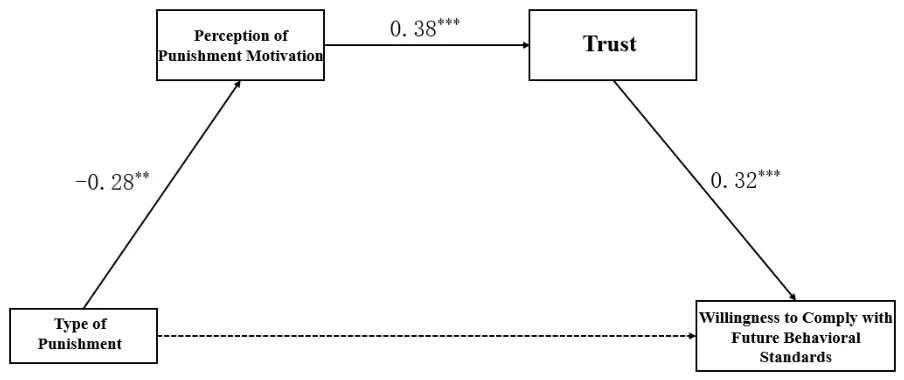
Path Diagram of the Chain-Mediated Model. ** *p* < 0.01, *** *p* < 0.001.

The results of the chained mediation analysis are presented in Table 4. The results indicate that the type of punishment significantly and negatively predicts perceived punishment motivation (*β* = −0.28, *p* < 0.01); perceived punishment motivation significantly and positively predicts trust (*β* = 0.38, *p* < 0.001); trust significantly and positively predicts the intention to comply with behavioral norms in the future (*β* = 0.32, *p* < 0.001), while the direct effect of punishment type on the intention to comply with behavioral norms in the future is not significant (*β* = −0.26, *p* > 0.05). The total effect of punishment type on future willingness to comply with behavioral norms was significant, *effect* = −0.47 (95% CI = [−0.71, −0.22], excluding 0); the direct effect of punishment type on future willingness to comply with behavioral norms was not significant, *effect* = −0.26 (95% CI = [−0.53, 0.22], including 0); the indirect effect of the path Punishment Type → Perceived Punishment Motivation → Trust → Willingness to Comply with Behavioral Norms in the Future was significant, *effect* = −0.03 (95% CI = [−0.07, −0.01], excluding 0), indicating that the full mediating effect of perceived punishment motivation and trust on the relationship between punishment type and willingness to comply with behavioral norms in the future reached statistical significance (see Figure 5). (see Figure 5). This provides support for Hypotheses 2b. Similarly, the chained mediating effect of perceived punishment motivation and trust on the relationship between punishment type and willingness to comply with behavioral norms in the future was statistically significant but modest in magnitude.

### 3.5. Moderated Mediation Analysis

**Figure 6:**
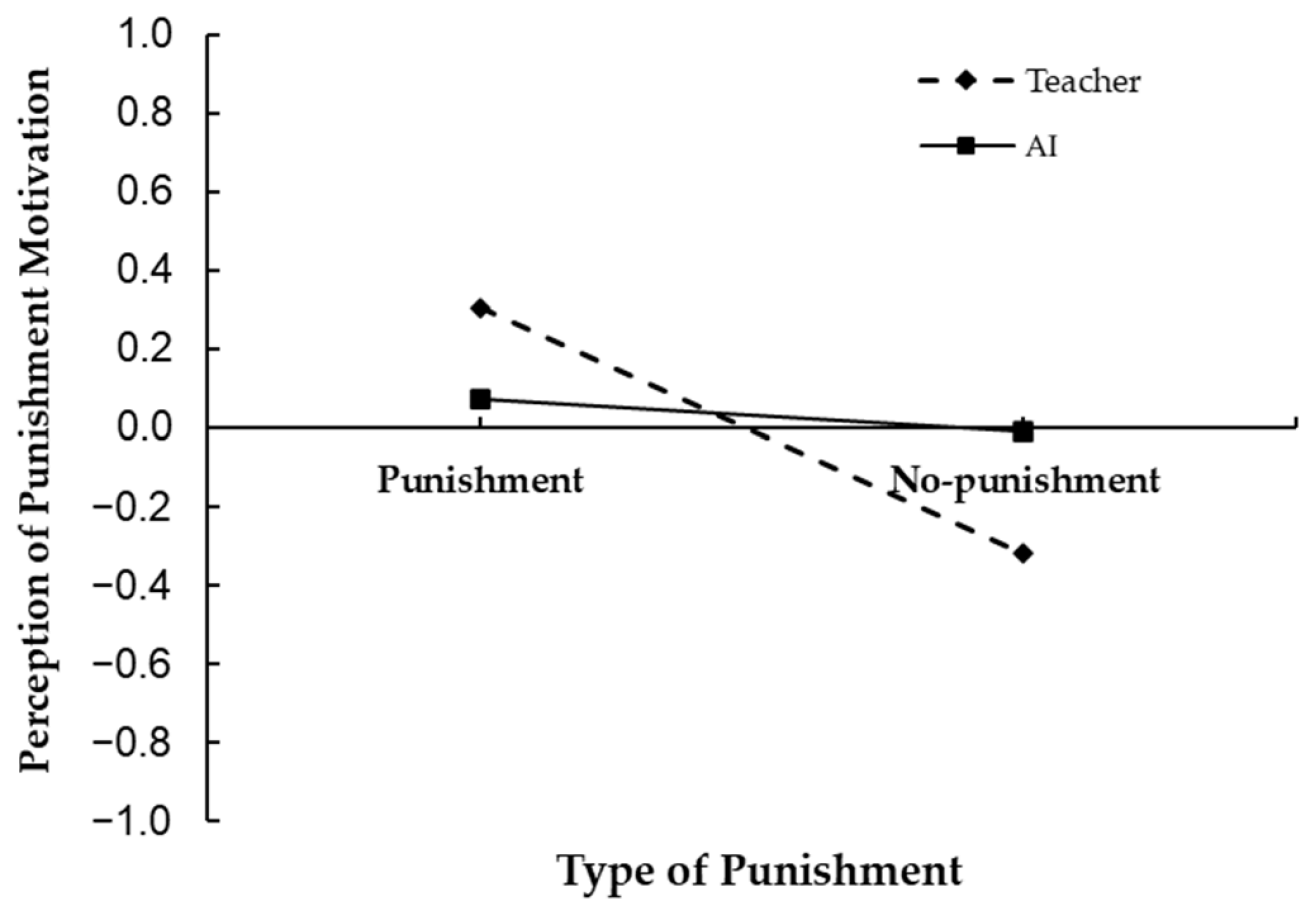
Simple slope plot of the interaction between punishment type and group relationship on perceived punishment motivation.

**Figure 7:**
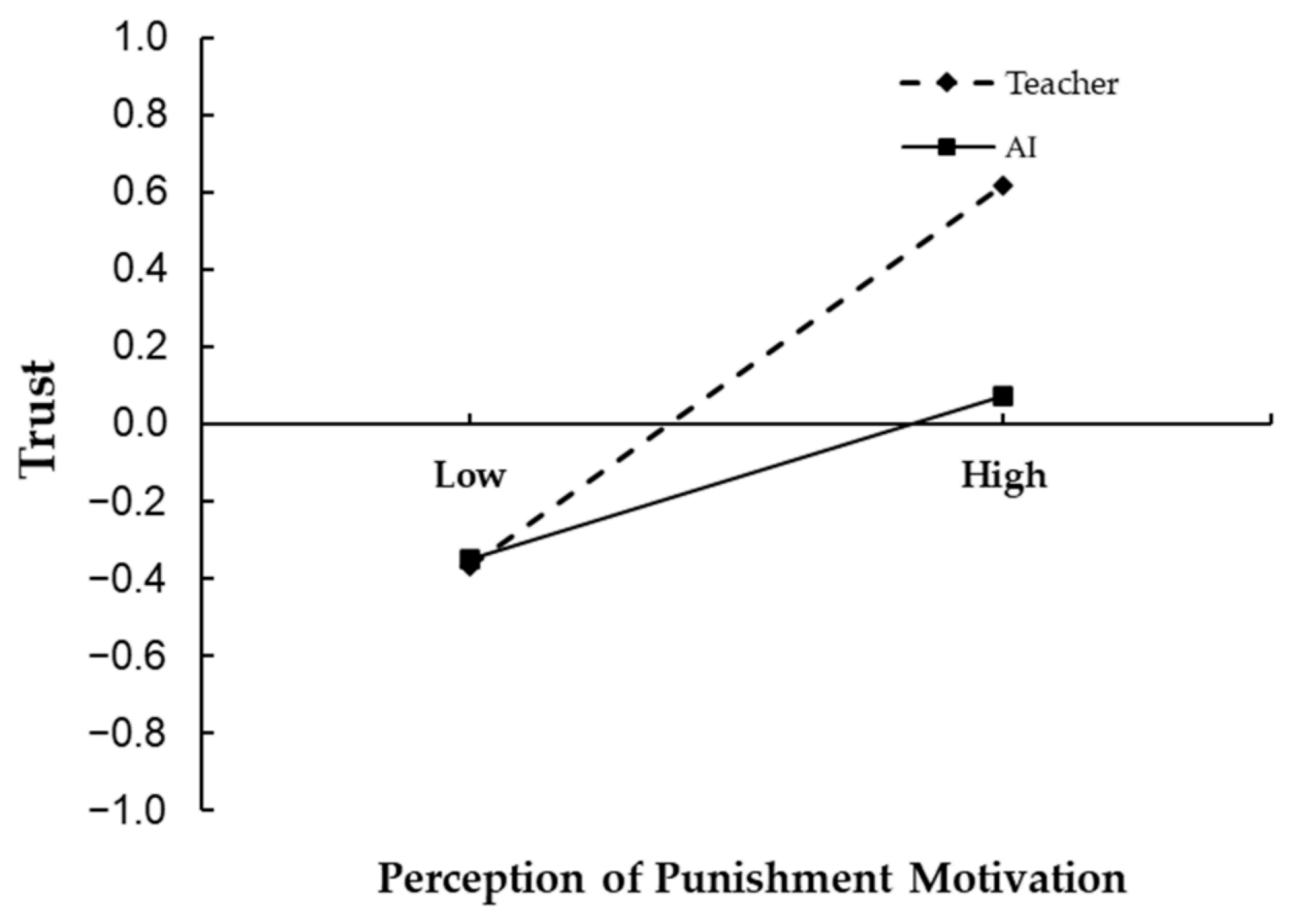
Simple slope plot of the interaction between group relationship and perceived punishment motivation on trust.

Building upon the mediation model, we incorporated group relations as a moderator to construct a moderated mediation model. First, using PROCESS Model 1, we tested the moderating effects of group relations on the relationship between punishment type and perceived punishment motivation, as well as on the relationship between perceived punishment motivation and trust; the results are shown in Table 5. Group affiliation moderated the effect of punishment type on perceived punishment motivation (*β* = 0.43, *p* < 0.05), as shown in Figure 6. Group affiliation moderated the effect of perceived punishment motivation on trust (*β* = −0.38, *p* < 0.001), as shown in Figure 7.

A simple slope test further indicates (see Table 6) that when the punisher is a teacher (in-group), the negative prediction of punishment type on perceived punishment motivation is significant (*β* = −0.49, *p* < 0.001), whereas when the punisher is an AI (out-group), the negative prediction of punishment type on perceived punishment motivation is not significant (*β* = −0.06, *p* > 0.05). Similarly, when the punisher is an AI (out-group), the positive prediction of perceived punishment motivation on trust is significant (*β* = 0.23, *p* < 0.01), whereas when the punisher is a teacher (in-group), the positive predictive effect of perceived punishment motivation on trust is significantly enhanced (*β* = 0.62, *p* < 0.001).

Next, we used PROCESS Model 58 to further examine the moderating role of group relationship in the model where perceived punishment motivation mediates the effect of punishment type on trust. The results indicate that group relationship moderates the effect of punishment type on trust via perceived punishment motivation, *effect* = −0.75 (95% CI = [−0.91, −0.59], excluding 0). Specifically, when the punisher was a teacher (in-group), the mediating effect of perceived punishment motivation was stronger, *effect* = −0.24 (95% CI = [−0.43, −0.08], excluding 0), whereas when the punisher was an AI (out-group), the mediating effect of perceived punishment motivation was not significant, *effect* = −0.01 (95% CI = [−0.08, 0.04], including 0). Providing support for Hypotheses 3.

To examine the moderating effect of group relationship on the chained mediation between perceived punishment motivation and trust, an analysis was conducted using PROCESS Model 92. As shown in Table 6, the results indicate that group relationship moderated the chain-mediated effect of punishment type on the willingness to change current rule-breaking behavior, *effect* = 0.05 (95% CI = [0.01, 0.12], excluding 0), and the chain-mediated effect of punishment type on the willingness to comply with behavioral norms in the future, *effect* = 0.02 (95% CI = [0.01, 0.06], excluding 0). Specifically, when the punisher is a teacher (in-group), the chained mediating effect of punishment type on the willingness to change current rule-breaking behavior through perceived punishment motivation and trust is stronger, *effect* = −0.06 (95% CI = [−0.12, −0.01], excluding 0). In contrast, when the punisher is an AI (out-group), the chain-mediated effect of perceived punishment motivation and trust is not significant, *effect* = −0.00 (95% CI = [−0.04, 0.02], including 0). Similarly, when the punisher is a teacher (in-group), the effect of punishment type on the willingness to comply with behavioral norms in the future, mediated through perceived punishment motivation and trust, is stronger, *effect* = −0.04 (95% CI = [−0.09, −0.01], excluding 0). These findings provide support for Hypothesis 4.

## 4. Discussion

The primary objective of this study is to examine whether perceived punishment motives and trust play a chained mediating role between punishment type and willingness to change behavior, with a particular focus on the moderating effect of group relationships. Correlation analysis revealed significant correlations between punishment type and both perceived punishment motivation and trust. Furthermore, mediation analysis indicated that perceived punishment motivation significantly mediates the relationship between punishment type and trust. Additionally, punishment type indirectly predicts students’ willingness to change behavior through the chained mediation of perceived punishment motivation and trust. Finally, moderation analysis revealed that group relationship moderates the relationship between punishment type and perceived punishment motivation, as well as the relationship between perceived punishment motivation and trust.

### 4.1. The Impact of Punishment Types on Students’ Willingness to Change Their Behavior

This study indicates that, compared to no punishment, punishment can influence students’ willingness to change their behavior, which is consistent with previous research (Jones et al., 2023; Iskandar et al., 2024; Yuda Mahendra et al., 2024). Specifically, when students engage in rule-breaking behavior, punishment is associated with higher willingness to change their behavior, whether regarding the current rule-breaking behavior or future compliance with behavioral norms, and students are more likely to accept the punishment imposed by teachers. This is also consistent with behaviorist theory, which posits that punishment, as an external consequence that reduces undesirable behavior, can promote prosocial behavior and clarify the boundaries of social norms (Skinner, 1965). Previous research on punishment has suggested that punishment is essentially a “double-edged sword”: when administered fairly, consistently, and with prosocial intentions, it may promote the internalization of norms and the building of trust (Mulder, 2008; Ho et al., 2019); however, punishment may also have negative effects. Some studies have indicated that, in the absence of guidance from social norms, punishment may actually undermine cooperation (Bicchieri et al., 2021). When punishment is too severe, students may associate their behavior with the punitive context or task. For example, students who are repeatedly punished for failing to complete math homework may develop an increasing aversion to math class, experience emotions such as anxiety and resistance, and even come to dislike their math teacher, leading to a deterioration in the teacher–student relationship (D.-J. Zhang, 2016). In accordance with educational principles, this study focuses on moderate punishment, viewing it as a form of evaluative feedback intended to facilitate communication; the results of this study also validate our hypothesis. As previous studies have found, for junior high school students, punishment can promote cooperative behavior in the public goods dilemma (Cui et al., 2017). Furthermore, research has shown that punishment can reduce various degrees of dishonest behavior among students (C. J. Liu et al., 2025), improve individual misbehavior such as inappropriate language, aggressive behavior, and defiance of authority (Ferrier et al., 2025), and correct erroneous behavior while enhancing positive performance (Calabrese et al., 2023). Cognitive psychology views negative feedback as a subtle form of punishment that expresses disapproval of an individual’s behavior (Kluger & DeNisi, 1996). Within constructivist approaches, negative feedback helps individuals actively learn and construct their understanding of what constitutes socially acceptable behavior (Vygotsky, 2011). Research has found that different forms of criticism from teachers toward students who violate rules can increase the closeness of the teacher–student relationship and enhance students’ willingness to improve their behavior (Cheng et al., 2025). Punishment can also promote positive behavior by suppressing negative behavior (Raihani & Bshary, 2019). These findings are consistent with the view that punishment, as a tool for deterrence and enforcement of norms, is associated with higher willingness to change behavior.

### 4.2. The Chain Mediation of Perceived Motivation for Punishment and Trust

The type of punishment influences students’ perception of punishment motivation prior to behavioral improvement. The study found that, compared to no punishment, punishing rule-breaking behavior enables students to perceive prosocial motives more strongly, thereby influencing their trust in the punisher. This is consistent with previous research, which shows that both punishment and feedback can influence individuals’ motivation and attitudes (Tricomi & DePasque, 2016), both of which are central to rule-breaking behavior, as motives drive behavior (Neal & Griffin, 2006). Furthermore, when individuals must pay a price for their misconduct by accepting a certain punishment, it triggers students to actively interpret the motives behind the punishment. According to attribution theory, when other cues are unclear, the punishment system is likely to serve as an external cue. On the one hand, it influences the attribution of others’ punitive behavior, thereby altering trust in others; on the other hand, it also influences the attribution of one’s own misconduct, thereby increasing the willingness to change non-compliant behavior (Martinko & Mackey, 2019). Furthermore, punishment conveyed with respect enhances the offender’s perception that the punisher is attempting to repair their relationship with the group (prosocial punishment motivation), reduces the perception of harm-oriented and self-serving motivations, and thereby increases trust in the punisher (de Vel-Palumbo et al., 2023). Previous studies have found that both monetary and social punishments influence individuals’ perceptions of the punisher’s motives, thereby affecting their trust in others (X. Liu et al., 2010). Some scholars argue that in second-party punishment scenarios, the punisher may incur certain costs, such as retaliation from the rule-breaker (Balliet & Van Lange, 2013). Consequently, when individuals perceive that the motivation for punishment is solely to prevent rule-breaking rather than to inflict harm, their trust in the punisher increases (Deutchman et al., 2021). Likewise, in this study, students are expected to perceive a higher level of prosocial motivation in the punishment they receive for their own rule-breaking, which in turn influences their trust in the punisher.

Punishment influences trust through the perception of punishment motivation, which in turn affects the offender’s willingness to change their behavior. The results of this study indicate that, compared to no punishment, punishment is associated with higher trust in the punisher, which is associated with a greater willingness to improve one’s misconduct. This is consistent with some existing research findings. For instance, studies have shown that student observers trust teachers more when they impose penalties for rule-breaking (Z. Zhang & Qi, 2024). When teachers are willing to manage disciplinary issues in the classroom, students’ trust in them increases, thereby fostering stronger teacher–student bonds (Simões & Calheiros, 2019). The higher the students’ trust in their teachers and the stronger the teacher–student bond, the more likely it is to curb students’ negative behaviors in the school environment, such as peer aggression, and the more likely it is to help students develop a greater willingness to exhibit positive social behavior within a group (Ettekal & Shi, 2020; Krause & David Smith, 2023).

In addition, this study found that punishment can trigger offenders’ perception of the punisher’s motives; when offenders perceive a high level of prosocial motivation behind the punishment, they report higher trust in the punisher, making them more willing to accept the punisher’s decisions and thereby more inclined to change their behavior. This is consistent with previous research on trust, which indicates that trust can promote various forms of prosocial behavior (Malti et al., 2016). When students trust their teachers, they are more likely to adhere to classroom norms and exhibit fewer disciplinary and aggressive behaviors (Krause & David Smith, 2023). When teachers manage students’ misbehavior (through criticism, punishment, etc.) while demonstrating understanding and support for students’ psychological needs, students perceive “goodwill” from the teacher, leading them to trust the teacher more and making them more likely to adjust their behavior intentions (Jiang et al., 2019). While this study found that the chained mediating effects of perceived punishment motivation and trust were statistically significant, the magnitude of these indirect effects was relatively modest. This also means that willingness to change behavior is likely influenced by a wide range of factors beyond punishment type and its downstream psychological processes, such as individual differences (e.g., personality traits, prior experiences with punishment), social influences (e.g., peer norms, family expectations), and contextual factors (e.g., school climate, teacher–student relationship quality). The findings of this study are consistent with the Theory of Planned Behavior. According to this theory, an individual’s behavioral intention is the most direct precursor to actual behavior, and behavioral intention is influenced by the individual’s attitude toward that behavior. Attitudes, in turn, stem from an individual’s cognitions and beliefs regarding the consequences of the behavior. The chained mediation model in this study, which specifies perceived motivation for punishment (cognitive attribution) to trust (attitude) and ultimately to willingness to change behavior (behavioral intention), has a structural correspondence with the theoretical hierarchy of the Theory of Planned Behavior (TPB). Punishment, as an external stimulus, is first cognitively processed by the individual (attribution: Is this punishment motivated by prosocial intentions?), and this cognitive outcome shapes the student’s attitude toward the punisher (trust or distrust), with the attitude ultimately influencing the student’s behavioral intention (willingness to change the rule-breaking behavior). This study effectively embeds the TPB’s “cognition to attitude to behavioral intention” framework into the specific social interaction context of a “punishment scenario,” revealing how external norm enforcement influences individuals’ willingness to change their behavior through the internal psychological processes described by the TPB.

### 4.3. The Moderating Role of Group Relationship

The results indicate that group relationship moderates the relationship between punishment type and perceived punishment motivation, as well as the relationship between perceived punishment motivation and trust. Specifically, when the punisher belongs to the in-group (teachers), punishment type exerts a stronger influence on trust through perceived punishment motivation, and punishment type exerts a stronger influence on students’ willingness to change their behavior through the chain mediation of perceived punishment motivation and trust. The findings of this study also validate Social Identity Theory, which posits that individuals tend to identify with the groups to which they belong, deriving a sense of belonging from this identification. When individuals have a strong sense of identification with a group, they are more likely to internalize the group’s norms and values. Previous studies have found that individuals perceive punishments from their in-group as more fair and reasonable; even when the punishment itself is threatening, this in-group relationship can buffer the negative effects of the punishment (Green et al., 2005). In addition, higher levels of trust are established among in-group members; this trust encompasses not only competence trust but also integrity trust and benevolence trust. When trust levels are high, it promotes information sharing and cooperative behavior among individuals (Gilgan & Titz, 2025). Consequently, within in-group relationships, even when faced with punishment, individuals are more likely to interpret it as constructive feedback, making them more likely to report a willingness to change non-compliant behavior and adhere to future norms (Moin et al., 2025). Some studies have compared feedback from computers or AI with feedback from humans, finding that participants perceived negative feedback from AI as less fair than that from humans (Thuillard et al., 2022). Another study indicated that AI feedback is less accepted than feedback from human supervisors, and that negative feedback provided by AI (criticism, punishment, etc.) makes employees feel unwelcome, thereby reducing their performance levels (Hein et al., 2024). This is also consistent with the findings of this study: compared to AI, students trust punishments imposed by teachers more and are more willing to accept teachers’ suggestions that correspond to greater self-reported willingness to change behavior.

It is also important to acknowledge that the teacher–AI distinction involves multiple dimensions beyond social categorization, including perceived humanness, empathy, pedagogical authority, familiarity, and competence. The present findings do not isolate group relationships from these human-vs-AI differences. Rather, we interpret the observed pattern as consistent with a social categorization framework, in which teachers—as human in-group members—elicit more positive attributions and trust than AI systems—as non-human out-group entities. Future research is needed to disentangle the specific contributions of these dimensions to the moderating effects observed here.

## 5. Limitations and Future Prospects

This study also has certain limitations. First, it focuses on only one type of disciplinary situation; future research could replicate and validate these findings using a larger sample and in a broader range of school settings. Furthermore, this study examines only the impact of public self-criticism on individual psychological reactions. However, educational disciplinary practices involve various forms of punishment, such as point deductions and parent-teacher conferences. These different forms of punishment vary significantly in terms of severity and the degree of social humiliation they entail, which may trigger different attribution processes and attitudinal responses. Furthermore, the situational experiment employed in this study relies on participants’ self-reported behavioural intentions after reading hypothetical scenarios, rather than on actually observed behavioural changes. This lack of real-world experience may lead to a stronger social desirability bias in the measurement of the dependent variable (future behavioral change). Some studies have found that the predictive power of trust regarding behavioral change following punishment fluctuates over time; future research could incorporate longitudinal tracking or field experiments to further observe changes in students’ behavior after they have been punished (Amemiya et al., 2020). Finally, this study operationalized group relationship as the social identity of the punisher (teacher vs. AI); however, the concept of “in-group and out-group” is inherently hierarchical and context-dependent, and students’ emotional evaluations of teachers may vary across different cultural or educational contexts. This study found that the punitive effect under the AI condition was weaker than under the teacher condition; this conclusion may apply only to the specific context of this study, in which AI served as an institutional authority figure. Future research should further compare the effects of AI on individual psychology and behavior across different roles. Furthermore, differences between teachers and AI may also involve dimensions such as perceived empathy, pedagogical authority, and competence; these factors may be specific manifestations of the in-group/out-group classification effect. Future research could further explore the specific roles these dimensions play in the in-group/out-group effect.

## 6. Conclusions

This study reached the following conclusions: perception of punishment motivation partially mediates the relationship between punishment type and trust; that is, compared to no punishment, when teachers punish students who violate rules, this is associated with higher levels of students‘ perception of the teacher’s prosocial punishment motivation. Furthermore, the perception of punishment motivation and trust exert a chained mediating effect between the type of punishment and students’ self-reported willingness to change behavior. Specifically, rule-breaking students’ perception of the teacher’s prosocial punishment motivation is positively associated with their trust in the punishing teacher, which in turn corresponds to greater self-reported willingness to change their behavior (including the willingness to change current rule-breaking behavior and the willingness to comply with behavioral norms in the future). It is important to note that these indirect effects, while statistically significant, were modest in magnitude. Nevertheless, even small effects can have meaningful practical implications. In educational settings, where numerous factors simultaneously shape student behavior, consistent small effects can accumulate over time and across multiple interactions to produce meaningful changes in student outcomes. However, these pathways are moderated by group relationship: when the punisher is a teacher, who belongs to the rule-breaking students’ in-group, punishment shows a stronger positive association with trust through the perception of prosocial punishment motivation, and also shows a stronger positive association with students’ self-reported willingness to change their behavior through both the perception of prosocial punishment motivation and trust, whereas when the punisher is an AI, which represents the out-group for the rule-breaking students, this indirect effect is not significant. These findings are consistent with a social categorization interpretation. However, given that teachers and AI systems also differ on dimensions such as perceived humanness, empathy, pedagogical authority, familiarity, and competence, the present results should be interpreted with appropriate caution, and future research is needed to further disentangle these dimensions. These findings reveal the psychological pathway through which punishment influences self-reported willingness to change behavior via sequential processing of cognitive attribution (perceived punishment motivation) and attitudinal evaluation (trust), and highlight the moderating role of group relationship in this process.

It is important to emphasize that the outcome variable in this study was participants’ self-reported willingness to change their behavior in response to hypothetical scenarios, rather than objectively observed behavioral change. As such, the findings pertain to reported behavioral intentions rather than actual behavior modification, and should be interpreted within the scope of this design.

## Figures and Tables

**Table 1:** Descriptive statistics and correlation analysis among variables.

Variable	*M*	*SD*	1	2	3	4	5
1. Type of Punishment	0.52	0.50	1				
2. Group Relationship	0.50	0.50	−0.05	1			
3. Perception of Punishment Motivation	2.72	0.79	−0.17 **	0.03	1		
4. Trust	2.59	0.82	−0.52 **	−0.11	0.45 **	1	
5. Willingness to change behavior	3.00	0.92	−0.29 **	−0.39 **	−0.01	0.28 **	1

Note: The penalty type is a dummy variable (Punishment = 0, No-Punishment = 1); the group relationship is a dummy variable (Teacher = 0, AI = 1); N = 240; ** *p* < 0.01; the same applies below.

**Table 2:** Regression Analysis of Various Variables on Trust.

Outcome Variable	Predictor Variables	*R* ^2^	*F*	*β*	*t*	95% CI
Trust	Type of Punishment	0.28	30.17 ***	−0.87	−9.54	[−1.05, −0.69]
Perception of Punishment Motivation	Type of Punishment	0.04	3.14 *	−0.28	−2.79 **	[−0.48, −0.08]
Trust	Type of Punishment	0.41	41.25 ***	−0.76	−9.06 ***	[−0.93, −0.60]
	Perception of Punishment Motivation			0.38	7.25 ***	[0.28, 0.49]

* *p* < 0.05, ** *p* < 0.01, *** *p* < 0.001.

**Table 3:** Regression Analysis of the Effects of Various Variables on the Willingness to Change Current Non-Compliant Behavior.

Outcome Variable	Predictor Variables	*R* ^2^	*F*	*β*	*t*	95% CI
Perception of Punishment Motivation	Type of Punishment	0.04	3.84 **	−0.28	−3.48 **	[−0.48, −0.08]
Trust	Type of Punishment	0.41	41.25 ***	−0.76	−9.06 ***	[−0.93, −0.60]
	Perception of Punishment Motivation			0.38	7.25 ***	[0.28, 0.49]
Willingness to change current non-compliant behavior	Type of Punishment	0.14	7.37 ***	−0.36	−2.63 **	[−0.63, −0.09]
	Trust			0.29	3.19 **	[0.11, 0.47]

** *p* < 0.01, *** *p* < 0.001.

**Table 4:** Regression Analysis of the Effects of Various Variables on the Intention to Adhere to Future Behavioral Norms.

Outcome Variable	Predictor Variables	*R* ^2^	*F*	*β*	*t*	95% CI
Perception of Punishment Motivation	Type of Punishment	0.04	3.15 **	−0.28	−2.79 **	[−0.48, −0.08]
Trust	Type of Punishment	0.41	41.25 ***	−0.76	−9.06 ***	[−0.93, −0.60]
	Perception of Punishment Motivation			0.38	7.25 ***	[0.28, 0.49]
Willingness to Comply with Future Behavioral Standards conduct	Type of Punishment	0.12	6.43 ***	−0.26	−1.82	[−0.53, 0.02]
	Trust			0.32	3.19 ***	[0.13, 0.50]

** *p* < 0.01, *** *p* < 0.001.

**Table 5:** Test of the Moderating Effect.

	Perception of Punishment Motivation			Trust		
	*β*	*t*	95% CI	*β*	*t*	95% CI
Type of Punishment	−0.49	−3.47 ***	[−0.77, −0.21]			
Type of Punishment × Group Relationship	0.43	2.12 *	[0.03, 0.83]			
Perception of Punishment Motivation				0.62	8.29 ***	[0.47, 0.77]
Perception of Punishment Motivation × Group Relationship				−0.38	−3.15 ***	[−0.62, −0.14]
Teacher	−0.49	−3.47 ***	[−0.77, −0.21]	0.62	8.29 ***	[0.47, 0.77]
AI	−0.06	−0.45	[−0.34, 0.21]	0.23	2.46 **	[0.04, 0.42]

* *p* < 0.05, ** *p* < 0.01, *** *p* < 0.001.

**Table 6:** Presents the test for moderated chain mediation.

	Willingness to Change Current Non-Compliant Behavior			Willingness to Comply with Future Behavioral Standards		
	*Effect*	*SE*	95% CI	*Effect*	*SE*	95% CI
Chain mediation effect	0.05	0.03	[0.01, 0.12]	0.02	0.01	[0.01, 0.06]
Moderating effect						
Teacher	−0.06	0.02	[−0.12, −0.01]	−0.04	0.02	[−0.09, −0.01]
AI	−0.00	0.01	[−0.04, 0.02]	−0.02	0.01	[−0.05, −0.01]
Comparison	0.05	0.03	[0.01, 0.12]	0.02	0.01	[0.01, 0.06]

Note: The chain of mediating effects is as follows: type of punishment → perceived punishment motivation → trust → willingness to change current non-compliant behavior; and type of punishment → perceived punishment motivation → trust → willingness to comply with future behavioral norms. Comparison: AI condition vs. teacher condition.

## Data Availability

The original contributions presented in this study are included in the article. Further inquiries can be directed to the corresponding author.

## Appendix A

The following comprises the situational materials used in this study.

**Teacher Punishment Condition:** Today it was your turn to be on duty, responsible for cleaning the classroom, but because you left in a hurry, you forgot to wipe the dust off the windowsills, and some paper scraps were left on the floor. As a result, your class lost points from the Student Council officer who inspected the classroom. Discipline issues in your class have always been handled by your experienced homeroom teacher, Mr. Li. Mr. Li has 10 years of experience teaching classes and has dealt with all kinds of student disciplinary issues. Your unsatisfactory cleaning duty record was reported to Mr. Li, who did not ask if you had any special circumstances and punished you by requiring you to make a public self-criticism in front of the class.

**Teacher No-Punishment Condition:** Today it was your turn to be on duty, responsible for cleaning the classroom, but because you left in a hurry, you forgot to wipe the dust off the windowsills, and some paper scraps were left on the floor. As a result, your class lost points from the Student Council officer who inspected the classroom. Discipline issues in your class have always been handled by your experienced homeroom teacher, Mr. Li. Mr. Li has 10 years of experience teaching classes and has dealt with all kinds of student disciplinary issues. Your unsatisfactory cleaning duty record was reported to Mr. Li, but he did not ask if you had any special circumstances, nor did he impose any punishment on you.

**AI Punishment Condition:** Today it’s your turn to be on duty, and you’re responsible for cleaning the classroom. However, because you left in a hurry, you forgot to wipe the dust off the windowsills, and some paper scraps were left on the floor. As a result, your class lost points from the student council officer who inspected the classroom. Your class has adopted the “AI System for Intelligent Student Behavior Management,” which is currently being piloted at the school. This system is powered by artificial intelligence and contains student management data from the past 10 years. Your record of failing to fulfill your cleaning duty was entered into the AI system, which made its final decision based on built-in school rules and regulations and penalties for similar cases. The AI system did not collect any information about this specific incident and imposed the following punishment on you: to publicly make a self-criticism in front of the class.

**AI No-Punishment Condition:** Today it’s your turn to be on duty, and you’re responsible for cleaning the classroom. However, because you left in a hurry, you forgot to wipe the dust off the windowsills, and some paper scraps were left on the floor. As a result, your class lost points from the Student Council officer who inspected the classroom. Your class has adopted the “AI System for Intelligent Student Behavior Management,” which is currently being piloted at the school. This system is powered by artificial intelligence and contains student management data from the past 10 years. Your record of failing to fulfill your cleaning duty was entered into the AI system, which makes its final decision based on built-in school rules and regulations and penalties for similar cases. The AI System for Intelligent Student Behavior Management did not collect any information regarding this incident, nor did it impose any punishment on you.
